# Managerial approaches for maintaining low levels of sick leave: A qualitative study

**DOI:** 10.1111/jonm.13678

**Published:** 2022-05-30

**Authors:** Sara L. Fallman, Lotta Dellve, Agneta Kullén Engström

**Affiliations:** ^1^ Faculty of Caring Science, Work Life, and Social Welfare University of Borås Borås Sweden; ^2^ School of Engineering Sciences in Chemistry, Biotechnology, and Health KTH Royal Institute of Technology Stockholm Sweden; ^3^ Department of Sociology and Work Science Gothenburg University Gothenburg Sweden

**Keywords:** employee, first‐line manager, flexibility, health care, sick leave

## Abstract

**Aim:**

The aim of this study was to identify first‐line managers' approaches for maintaining low levels of sick leave among health care employees.

**Introduction:**

One challenge in health care is the high level of sick leave among employees. High work demands and conflicting pressures characterize the work situation of both employees and first‐line managers, with potential negative effects on work‐related health.

**Method:**

First‐line managers at units with low and/or decreasing sick leave were interviewed. Thematic analysis was used to analyse the data.

**Results:**

The managers took a holistic approach in meeting their employees' broader needs, and they were balancing high organisational demands through insubordination. To keep sick leave rate low, they created possibilities for the employees to influence their own working life through a present, visible and trustful leadership.

**Conclusion:**

Managers responsible for units with low sick leave seemed to utilize a holistic approach with focus on their employees and prioritized needs of their employees before organisational demands from top management.

**Implications for nursing management:**

First‐line managers in health care can have impact on sick leave among their employees and create good working conditions, despite pressure from their superiors.

## 1 INTRODUCTION

One challenge in health care today is high numbers of sick leave among health care workers. Many studies highlighting this phenomenon focus on conditions associated with high levels of sick leave among employees. Few studies investigating reasons for low sick leave in organisations explicitly focus on the public health care sector. Most research on the topic has been conducted in private companies and the public sector in general (Ljungblad et al., 2014; Stoetzer et al., 2014).

The current study focuses on the approach of first‐line managers in health care units with low levels of sick leave. Sick leave and illnesses among employees have increased across Sweden during the last decade, especially among health care employees with a peak in 2016 (Swedish Social Insurance Agency, 2018). Persistent sick leave can have negative consequences, both physical and economic, for the employee. It also contributes to large societal costs (Swedish Social Insurance Agency, 2019). In addition to the high rates of sick leave and turnover among health care employees, there is also an upcoming shortage of health care employees. Shortages of employees in health care, especially nurses, are reported from around the world, for instance, the UK (Beech et al., 2019), the US (Bureau of Labor Statistics, 2020) and Canada (CNA, 2020). This situation could have far‐reaching consequences and needs to be addressed.

Health care employees today have a workplace environment that is often characterized by high pressure, fast work pace and high demands (Aiken et al., 2013; Duffield, Diers, et al., 2011), which can mean that they do not have the time to perform all their work tasks. This may trigger a chain of negative outcomes, including stress, burnout and, in the long term, extended sick leave. Studies have shown a connection between work overload and sick leave among nurses (Rauhala et al., 2007). In turn, sick leave and a shortage of employees result in a higher workload for the employee at work, which will affect the quality of care (Duclay et al., 2015). In order for employees to achieve a sustainable work–life balance, flexible working conditions have become more common during the last decades. Joyce et al. (2010) showed that flexible working arrangements, such as flexitime, aimed at increasing employees' control had a positive impact on employee health. Other studies have reported that opportunities to influence and adjust their work tasks have been linked to not only a more sustainable return to work after long‐term sick leave (Dellve et al., 2016). On the other hand, low opportunity to influence work had a negative impact on sick leave (Hultin et al., 2010). The benefits of flexibility at work, providing autonomy and control to better meet work demands are in line with mechanisms of workplace resources that are explained through the job demand resource model (Bakker & Demerouti, 2007).

The work situation of first‐line managers in the health care sector is complex and multifaceted (Pegram et al., 2015) with diverse role responsibilities and competing demands (Baker et al., 2012) from different institutional logics and often without sufficient resources (Udod et al., 2017). Line managers additionally have to interact with many different professional groups as well as patients and their relatives, which increase their work complexity (Wong & Laschinger, 2015). They also play an important role for the quality of care and employee and patient outcomes (Boamah et al., 2018; Cummings et al., 2018; Fuller, 2015). Despite their considerable responsibilities, line managers often have limited decision‐making authority because of strict top‐down management control. At the same time, they have the overall responsibility for the unit's finances, which has been reported as being associated with reduced health, extensive over time and decreased ability to perform their work for first‐line managers in health care (Fallman et al., 2019; Kath et al., 2012).

Studies have shown connections between nurse managers' leadership style and health‐related outcomes among their employees, such as work satisfaction, performance, intention to quit (Cummings et al., 2018; Saleh et al., 2018) and levels of sick leave (Schreuder et al., 2011). Other studies have shown positive associations between beneficial working conditions and improved social environment (Strömgren et al., 2017). A trustful leadership with a visible and present manager has been associated to employee satisfaction in previous studies (Duffield, Roche, et al., 2011) and fewer days of sick leave (Dellve & Fallman, 2020).

In sum, although there are many studies about sick leave among health care employees, few investigations have attempted to explain low levels of sick leave in relation to managerial approaches for handling organisational conditions. The focus of this study is, therefore, to identify common patterns in managerial approaches at health care units with low levels of sick leave.

## 2 AIM

The aim of this study was to identify first‐line managers' approaches for maintaining low levels of sick leave among their health care employees.

## 3 METHOD

### 3.1 Design

This study has a qualitative design. Interviews were performed with managers of units with low and/or decreasing rates of sick leave according to the hospital employee records. Thematic analysis was used to analyse the managers' approaches that may have had an impact on their health care employees' working conditions.

### 3.2 Sample and data collection

Two hospitals in the same region were included due to the existence of ethical approval. The sampling included managers with experience of leading a health care unit with low and/or decreasing levels of sick leave. The register‐based data concerning employees' sick leave per unit over a 4‐year period (2013–2016) were utilized. The total number of units in the data was 43. Among the units, 14 were categorized as having low (i.e., a mean of <3.8% of staff on sick leave in 2013) or decreasing sick leave among their workers (the mean sick leave being 8.7% in 2013 and 4.9% in 2016). The managers of these units were contacted first by email to inform them about the study and thereafter by phone about participation. Almost all managers (13 out of 14) agreed to participate; two were excluded because of the shorter time (<2 years) they had been a manager of the unit. The final study group consisted of 11 managers, 9 women and 2 men, with 2–20 years' experience as head of the unit in question, and all of them had a degree in a health science or medicine. The number of employees in these 11 units ranged from 8–61 (median being 35).

All interviews were conducted individually, face to face. The interviews were conducted by SLF or AKE and ranged between 50 and 90 min (*M* = 70 min).

### 3.3 Analysis

The interview data were analysed using thematic analysis according to the six steps of Braun and Clarke (2006). The interviews were transcribed verbatim, and then, the researchers separately read through the interviews several times. The first step was to read and become familiar with the data, and the researchers made notes in the margin. After a thorough reading of the texts, the second step was to generate the initial coding. The first three interviews were analysed and coded by both researchers together to ensure some inter‐rater reliability. The other interviews were coded individually by both researchers. This step also consisted of comparing and reaching consensus between the two authors' codes. The third step was to search for themes by analysing the coding in several steps. In the fourth and fifth steps, themes were reviewed and redefined into new themes. This was done in order to ensure that the themes worked in relation to the data. In the last step, the entire data were read in relation to the whole data set, as recommended by Braun and Clarke (2006). This was done to check if the themes worked in relation to both the codes and the data set. An example of the process is illustrated in Table 1.

**TABLE 1:** Example of data extraction, codes, subtheme and theme

Data extraction	Codes	Subtheme	Theme
I am expected to report to the administration but have learned to prioritize and only submit some work. It is not only out of dissatisfaction but also to maintain my health.	Prioritize what to report Maintaining health	Neglecting administrative demands	Balancing high organisational demands through insubordination

## 4 RESULTS

This study highlights that managers in today's health care settings are facing demands that could be contradictory. On one hand, they need to meet the demands of the organisation and top management while simultaneously trying to meet the demands from their employees to create sustainable working conditions. The analysis of the data resulted in two themes: (1) a holistic approach to meet the needs of employees and (2) balancing high organisational demands through insubordination. The first theme related to the managers' relationships to the employees. The second theme concerned the managers' strategies in handling the effect of demands to create sustainable working conditions for both themselves and their employees.

An overview of the identified themes and subthemes is presented in Table 2.

**TABLE 2:** Themes and subthemes

Themes	Subthemes
Holistic approach to meet the needs of employees	Flexibility regarding employees' working conditions Flexibility regarding employees' personal situations Being present and visible as a manager Trustful relations
Balancing high organisational demands through insubordination	Exceeding the financial constraints Ignoring high demands for productivity Neglecting administrative demands

### 4.1 Holistic approach to meet the needs of employees

The first‐line managers seemed to have a genuine interest in other people. A holistic approach for them included considering the whole person and their situation both at and outside work. This requires flexibility regarding the working circumstances as well as supporting the employees and creating trustful relations.

#### 4.1.1 Flexibility regarding employees' working circumstances

Almost all interviewed managers put significant effort into meeting the employees' needs. They worked hard to approve vacation time or parental leave even at short notice. According to the managers, this was possible because their work schedules did not include full staff planning and scheduling and the employee was flexible and willing to help each other. They also gave the employees the option of working part‐time. The managers said that there were benefits to letting employees choose their own working hours. This also gave the managers the flexibility in how they staffed the unit.

We believe that if we let people choose their working hours, and they work 90% or 95%, maybe they can remain at work, and then we can hire one more employee. (P10)

The managers tried to develop special solutions for employees who wanted to adjust their workload and working hours. This gave the employees more possibilities to recover between shifts, and, thus, the managers felt this would decrease potential sick leave. Implementing this approach required flexibility, creativity and generosity according to the managers.

#### 4.1.2 Flexibility regarding employees' personal situations

The managers were also flexible regarding the employees' private life. They tried to see the situation as a whole and were aware that if someone had a stressful situation at home, it would reflect on that person's work. The managers pointed out that if they were not flexible and did not care about their employees' personal life, this could ultimately result in sick leave among their staff. Therefore, they offered the employees the possibility to take some leave if needed. The managers felt this strategy was successful in working towards a sustainable, healthy work–life balance.

Today, I, as a manager, can choose to say that this is your private problem and that I do not care. But it will not help because then I might lose the employee. (P11)

So they offered the employees the possibility to take some leave if needed. This strategy seemed to be successful in working towards a sustainable, healthy work–life situation.

#### 4.1.3 Being present and visible as a manager

Almost all of the managers described themselves as present and visible. They stated that they tried to be available to the employees and to be a part of the employees' daily work. Some said they made the effort to attend the unit meeting every morning. According to the managers, this presence signaled to the employees that they were accessible.

I am here early in the morning both to meet the night shift and to attend the morning meeting. I believe this promotes health and team spirit. (P6)

One manager stated she loved the summer because it gave her the opportunity to work bedside. In some units, the managers were physically absent but tried to compensate for geographical distance by being available by phone. The managers highlighted that a leadership style characterized by close interaction between managers and employees could prevent sick leave and facilitate the return to work after sick leave.

#### 4.1.4 Trustful relations

The managers highlighted the importance of trust and engagement between managers and employees and within the group. They emphasized that it was important to know their employees and were aware of their strengths and weaknesses. This enabled them to balance demands and tailor the tasks to each employee's abilities. They described the employees' generous attitude and acceptance of, and tolerance for, their colleagues' special needs on occasion. They emphasized the importance of paying attention to all employees and making them feel valued both at the workplace and away from it.

My employees know that I give them time off at short notice but that I also expect them to step up when needed. (P7)

The managers stated they worked continuously to maintain a good psychosocial working environment and applied their skills and effort to create trustful relations. At the same time, they did not emphasize their own leadership position. They were aware that a positive work environment with good staffing levels and a good psychosocial environment was fragile and could easily be broken, for example, due to staff shortages or unreasonable demands for cost savings.

### 4.2 Balancing high organisational demands through insubordination

In this second theme, the managers described that they used insubordination when they perceived the organisational demands were too high. They used this technique, such as ignoring requests, to protect both the employees and themselves. According to the managers, even if they used insubordination to maintain a balance at work, they perceived themselves as loyal to the organisation.

#### 4.2.1 Exceeding the financial constraints

According to the managers, one of the most important considerations in their daily work, set by top management, was finance. From the managers' perspective, they were constantly reminded of that budget controls everything. There were clear demands for a balanced budget, and when this was in jeopardy, the managers were expected to take action. Despite these expectations, the managers sometimes chose to break the rules and exceeded the budget to protect both their employees and themselves.

The managers argued that a factor that contributed to lower sick leave was that they were well staffed, which meant that the employees were not completely overwhelmed after a shift. The interviewed managers pointed out that superior manager sometimes told them that in order to keep balanced budget, they needed to reduce staffing hours. (P4)

I do not care if I overstaff because we have the right level of staff based on our production. The financial issues I have to deal with are on another level.

Although expected by top management, many managers chose not to inform their employees about the financial situation. According to the managers, a focus on finance was burdensome, like a heavy wet blanket, for most employees. The managers pointed out that they avoided discussing financial issues to protect the employees.

#### 4.2.2 Ignoring high demands for productivity

The first‐line managers described top management's high demands for efficiency and increased productivity under tight economic constraints. They described that they were constantly under pressure to increase productivity. At the same time, there were demands to maintain the quality of care: an equation they considered impossible. One strategy for handling this pressure was to ignore the organisational demands.

I mean it is not possible to twist and turn too much when there are many employees on sick leave. Instead, we cancel patient visits and, of course, that will be noticed in our productivity. (P3)

Another way of ignoring demands for increased productivity was to limit the inflow of patients. Most of the managers used this strategy. This option allowed the employees to perform their work without stress. The managers thought that this strategy would affect the sick leave and in the end increase productivity.

Another strategy the managers used to control employee workload was to refuse employees to take extra shifts in either their own or other units. At the same time, the managers did not hesitate to let employees from other parts of the organisation take an extra shift at their units.

#### 4.2.3 Neglecting administrative demands

The administrative support services at the hospitals have been centralized and reduced over time, which had adversely affected the amount of support the managers were given. Instead, the managers now were responsible for delivering data to the administrative support services. The managers pointed out that there was not enough time to deliver all the requested information. Their strategy for handling this situation was to either silently disregard follow‐up demands or openly refuse to deliver the requested information. They added that, in their opinion, administrative support staff could easily retrieve the data themselves. This was a way for managers to express resistance towards the system.

I do not report everything that is expected of me, and in the end, no one asks for it. It's a strategy for managing my own health and the work environment. (P2)

The participants criticized the limited support they were getting from the Human Resources (HR) department and said that administrative support had decreased considerably and that tasks that had previously been performed by HR had now been transferred to them as managers.

## 5 DISCUSSION

The main findings show that first‐line managers who were successful in maintaining low levels of sick leave share some common strategies. These strategies were characterized by a holistic approach to meet the employees' needs while balancing high organisational demands.

Managers in this study put a substantial amount of effort into meeting the needs of the employees to find a good work–life balance for the employee they are managing. This balance was achieved by providing flexibility in work hours, scheduling, vacations and work tasks. Based on the lower levels of taken sick leave in the studied units, these may be effective strategies to lower sick leave. To achieve better health‐related outcomes, having this type of balance between work and private life has also been highlighted in previous studies (Belton, 2018; Ejlertsson et al., 2018). The younger generations, who make up an increasingly larger proportion of the work force, prefer more flexible working conditions to a higher degree (Deloitte, 2019). At the same time, how far can a manager stretch to meet the employees' requests of flexibility? Is it acceptable that the organisations' come second?

Another factor that could have affected low sick leave was that the managers were present, visible and accessible to their employees. The managers in this study showed genuine presence, which was more than mere physical presence. A visible and present leader is able to get an understanding of the workload and make an overall evaluation of the situation at work, which is in line with the extant literature (Smith, 2013). Being present and visible gives managers the opportunity to detect early signs of decreased health among their employees (Crumpton, 2010).

In this study, one way of maintaining low levels of sick leave was to circumvent directives placed on them from upper management. This raises the question: Do we want these types of managers in health care, or do we prefer managers who always follow directives from above? Lack of alignment to top management's strategies may have a negative impact on the organisation but positive impact for the employees. Also, the importance of keeping to the budget and delivering all follow‐ups as requested can be questioned, and many managers in the study indicated they ignored them at times, giving them a certain amount of flexibility and balance at work. Despite the success of these strategies, managers may be considered disloyal if they do not follow the directives from top management. Furthermore, other studies have shown that the autonomy of first‐line managers has been reduced (Ericsson & Augustinsson, 2015). This lack of autonomy has been shown to be undesirable as top‐down management control has a negative effect on managers' self‐rated health and can have negative impacts on managers' abilities to perform managerial tasks (Fallman et al., 2019).

The first‐line managers' mission is complex. They have responsibility for the employees' health and wellbeing, are expected to keep sick leave low, stay within budget and maintain productivity. This creates competing demands, and managers experience being squeezed between the demands and expectations from top management and employees' needs.

### 5.1 Strengths and limitations of the study

One strength of the study is that data on sick leave at a unit level over a 4‐year period were used to select the managers for the interviews. Having longitudinal sick leave data over a 4‐year period also made it possible to investigate units with lower or decreasing levels of sick leave. This increases the validity of the study compared to a cross‐sectional selection. Another strength of the study is that the results are in line with theories of health promotion and organisational management. The inclusion criteria of 2 years in the managerial role was based on the assumption that during that time, the managers had gained enough experience to be able to answer the questions with credibility. A limitation of the present study can be that only two hospitals were included. On the other hand, the units included represented both prehospital, inpatient and outpatient care. The analyse method, thematic analysis, was chosen due to its flexible approach.

## 6 CONCLUSIONS

This study highlights some managerial strategies that were used to maintain a lower level of sick leave. In order to keep lower levels of sick leave among employees, the managers seemed to utilize a holistic approach towards their employees, seeing them as a person not only a worker. Furthermore, managers mentioned that prioritizing and creating a flexible work environment and developing trustful relations decreased the need for longer sick leave even if it meant being insubordinate towards organisational demands at times. These aspects need to be further investigated in order to reach a sustainable work life, and the results from this study could be used to trigger new research in the area.

### 6.1 Implications for nursing management

This study contributes to the knowledge about first‐line health care managerial strategies with the aim to maintain lower sick leave among employees. It shows that the managers were in a position to create good working conditions for their employees despite directions and pressure from top management. It is important to use units with lower sick leave as good examples to learn from them to create healthy and sustainable organisations. Hopefully, this study can inspire nurse managers to learn from units with low sick leave in order to create healthy and sustainable work organisations.

## CONFLICT OF INTEREST

The authors declare no conflict of interest.

## ETHICS STATEMENT

The study was approved by the Ethics Committee for Gothenburg Region, Sweden (Dnr. 1075‐16).

## Data Availability

Data available on request from the authors.
